# Evaluation of quality of life, sleep quality, depression, and nutritional status in community living older adults using videoconference method

**DOI:** 10.1097/MD.0000000000044478

**Published:** 2025-09-12

**Authors:** Özgün Elmas, Mustafa Cemali, Özge Cemali

**Affiliations:** a Department of Physiotherapy and Rehabilitation, Siirt University, Faculty of Health Sciences, Siirt, Turkey; b Department of Occupational Therapy, Trakya University, Faculty of Health Sciences, Edirne, Turkey; c Nutrition and Dietetics Department, Trakya University, Faculty of Health Sciences, Edirne, Turkey.

**Keywords:** depression, nutrition, older adults, quality of life, sleep quality, teleassessment, videoconference

## Abstract

The aim of this study was to evaluate quality of life, sleep quality, depression level, and nutritional status in older adults using a videoconference-based method, and to examine the effects of these variables on quality of life through a multiple linear regression model. Eighty-four older adults aged 65 to 75 with a mean age of 69.85 ± 2.34 participated in the study. Evaluations of older adults were made using the videoconference method via smartphone. Quality of life of older adults was assessed with the Nottingham Health Profile, sleep quality with the Pittsburgh Sleep Quality Index, depression status with the Geriatric Depression Scale-Short Form, and nutritional status with the Mini Nutritional Assessment-Short Form. According to the videoconference-based assessments, 58.3% of older adults were found to have poor sleep quality, 40.5% exhibited moderate or higher levels of depressive symptoms, and 36.9% were identified as malnourished. Multiple linear regression analysis revealed that sleep quality, depression, and nutritional status together accounted for approximately 71.9% of the variance in quality of life (*P* < .001). Among these variables, depression level showed the strongest standardized effect on quality of life (Beta = .902), followed by nutritional status (Beta = –.463) and sleep quality (Beta = .440). In this study, assessments conducted via videoconference revealed a high prevalence of sleep disturbances, depressive symptoms, and malnutrition among older adults. The findings demonstrated that sleep quality, depression, and nutritional status had strong effects on quality of life. The high proportion of explained variance (71.9%) highlights the importance of these interrelated factors. Together, these 3 variables explained 71.9% of the variance in quality of life, highlighting the critical role of psychological and behavioral factors in understanding quality of life in older adults. Moreover, this study suggests that videoconference-based assessment may serve as a practical, accessible, and time-efficient approach for remotely evaluating key health-related parameters in the older population.

## 1. Introduction

Older adults are rapidly increasing worldwide, and this demographic shift is having multidimensional impacts on health systems. As of 2023, approximately 14% of the world’s population is aged 60 and over, and this number has reached 1.1 billion. The World Health Organization predicts that this proportion will rise to 16% in 2030 and 22% in 2050, with the number of older adults reaching 2.1 billion.^[1,2]^ This increase in the proportion of older adults leads to an increasing prevalence of health-related physical, psychological, and social problems as life expectancy increases.^[3]^

Quality of life is a multidimensional construct that encompasses an individual’s physical functionality, cognitive status, psychological health, social relationships, and dietary habits.^[4]^ Recent studies have shown that poor sleep quality, depression symptoms, and nutritional deficiencies have a significant negative impact on quality of life. In a multicenter study, an 18% to 22% decrease in quality of life scores was reported in older adults with poor sleep quality.^[5]^ An increase in the level of depression was found to significantly reduce quality of life.^[6]^ It was determined that quality of life was 25% to 30% lower in individuals with inadequate nutritional status compared to those with adequate nutrition.^[7]^

With the COVID-19 pandemic, restrictions in access to healthcare services have increased the importance of digital health applications and popularized the use of video conferencing-based assessment methods.^[8]^ Video conferencing allows remote assessment of health status through synchronous or asynchronous communication between healthcare professionals and individuals.^[9]^ Previous studies have demonstrated that videoconferencing can be effectively utilized for the assessment of individuals within the pediatric population with cerebral palsy as well as those with neurological conditions such as Parkinson disease.^[10–12]^ However, as far as we know, there are no holistic studies on the evaluation of quality of life and these factors in older adults by video conferencing method. In addition, the existing literature generally examines the effects of these variables on quality of life separately, and studies in which multivariate regression analyses evaluating the effects of sleep quality, depression and nutritional status on quality of life together are limited.^[13,14]^ In this context, the aim of this study was to evaluate quality of life, sleep quality, depression level and nutritional status in older adults by video conferencing method and to examine the effect of these variables on quality of life with multiple linear regression model.

## 2. Methods

### 2.1. Study design

Permission was obtained for the study from the Lokman Hekim University Scientific Research ethics committee (Date: October 23, 2023, decision no: 2023/207). Online informed consent was obtained from older adults who participated in the study, and the study was conducted in accordance with the Declaration of Helsinki. The study took place between July and December 2024. Adults aged 65 to 75 years and older with no cognitive impairment participated in the study. The participants’ quality of life, sleep, depression, and nutritional status were evaluated via videoconference (online applications). The Strengthening the Reporting of Observational Studies in Epidemiology (STROBE) Checklist guidelines were followed for this study.^[15]^

### 2.2. Participants

The research sample consists of older adults who volunteered to participate in the study. Inclusion criteria were being 65 years of age or older, having no cognitive problems, being able to communicate, having access to a smartphone (either personally or through a relative), and having internet access. Amputees, those with serious chronic diseases that would affect functionality (psychiatric, cancer, neurological, orthopedic, rheumatic, cardiopulmonary, and systemic diseases), those unable to walk independently, and those who had undergone surgery in the last 6 months were considered as exclusion criteria. The sample size of the planned study was calculated using the G*Power 3.1.9.6 program, by selecting the correlation, point biserial model parameter from the F test family, and by using A priori type power analysis. Using the Nottingham Health Profile (NHP)^[16]^ as a reference, the minimum required number of participants was determined as 70 with a medium effect size (.36), 95% confidence interval, and 80% power. Participants were recruited using the snowball sampling method.^[17]^ Initially, contact was made with local associations, foundations, and community centers in Ankara, where older adults were known to be active members or regularly spend their time. Through these initial contacts, volunteer older adults who met the inclusion criteria were identified and invited to participate. Subsequent participants were then referred by those already enrolled in the study, allowing for the sample to expand progressively through peer networks. For the study, 90 people were evaluated via videoconference teleassessment method. Six participants were unable to complete the study due to internet connection interruptions. The study was completed with 84 older adults. All participants were community-dwelling older adults residing in Ankara, and all assessments were conducted in the Turkish language.

### 2.3. Data collection tools

A single videoconference session was scheduled per participant, with each session lasting approximately 30 to 50 minutes depending on the individual’s pace and internet stability. WhatsApp was used as the videoconferencing platform due to its accessibility and familiarity among older adults. During each session, a trained researcher read all questions and scale items aloud, and participants responded verbally. All responses were recorded manually by the interviewer in real-time.

### 2.4. Socio-demographic information form

Participants’ age, sex, height, and weight were obtained through a self-administered information form prepared by the researchers. Body mass index (BMI) was calculated using self-reported height and weight values.

### 2.5. NHP

It was developed by Hunt et al to assess the general health status and quality of life of individuals.^[18]^ NHP measures a general health status that measures the distress perceived by individuals in physical, emotional, and social areas. It consists of a total of 38 items: physical mobility (8 items), pain (8 items), sleep (5 items), emotional reactions (9 items), social isolation (5 items), and energy level (3 items). Each item is answered with “yes” or “no.” NHP allows a multi-faceted assessment of a person’s health status. The total score is scored between 0 to 500 and an increase in the score means a decrease in health status and quality of life. Turkish adaptation was made by Küçükdeveci et al and it was found that the Cronbach alpha coefficient of its sub-domains varied between .56 and .83.^[19]^

### 2.6. Pittsburgh Sleep Quality Index (PSQI)

PSQI was developed by Buysse et al to assess sleep quality.^[20]^ The scale determining sleep quality consists of self-report questions and evaluates sleep quality in the last 4 weeks. PSQI consists of 19 questions that evaluate 7 areas such as subjective sleep quality, sleep latency, sleep duration, achieved sleep efficiency, sleep disturbance, use of sleeping pills, and daytime dysfunction. The total score varies between 0 and 21, and an increase in the total score means a decrease in sleep quality. In addition, a total score of ≥ 5 indicates high sleep quality, and a score of < 5 indicates good sleep quality. The Turkish validity and reliability study of the scale was conducted by Ağargün et al Cronbach alpha coefficient was determined as .798.^[21]^ The PSQI has also been shown to be successfully applicable in telehealth practices conducted via video conferencing.^[22]^

### 2.7. Geriatric Depression Scale-Short Form (GDS-SF)

GDS-SF is a 15-question scale developed by Burke et al in 1991 to assess the level of depression in geriatric individuals.^[23]^ Participants are asked to respond “yes” or “no” to each question based on their condition during the past week. Each item is scored 1 point, and the total score is calculated by summing the points. Higher total scores indicate greater depressive symptoms. Scores ranging from 0 to 4 indicate no depressive symptoms, scores between 5 and 8 indicate mild depressive symptoms, scores between 9 and 11 indicate moderate depressive symptoms, and scores above 12 indicate severe depressive symptoms. The Turkish reliability and validity study of the scale was conducted by Durmaz et al, reporting a Cronbach alpha coefficient of .92.^[24]^ The GDS-SF has also been shown to be reliably administered through video-based telehealth assessments.^[25]^

### 2.8. Mini Nutritional Assessment-Short Form (MNA-SF)

MNA-SF was developed by Kaiser et al to evaluate nutritional status.^[26]^ The MNA-SF, used for screening malnutrition in older adults, consists of 6 items. The scale is scored based on the patient’s appetite changes, weight loss over the past 3 months, mobility during the last 3 months, psychological distress or acute illness, presence of neuropsychological problems, and BMI. An increase in the total score indicates an improvement in nutritional status. According to the MNA-SF scores, nutritional status is classified as normally nourished (11–14), at risk of malnutrition (7–10), or significantly malnourished (<7). The validity and reliability study of the MNA-SF in Turkish was conducted by Sarikaya et al.^[27]^

### 2.9. Data analysis

IBM SPSS Statistics 22 (Statistical Package Social Sciences, IBM Corp, Armonk) analysis program was used for statistical analyses. The normality test of the data was performed according to skewness–kurtosis, Kolmogorov–Smirnov, and histogram graphs and it was determined that the data were normally distributed. Descriptive variables were calculated as mean ± standard deviation for numerical data, number (n), and percentage (%) for categorical data. Multiple linear regression analysis was performed to examine the predictive relationships between variables and to evaluate the effects of sleep quality, depression status, and nutrition on quality of life. The statistical significance level was accepted as .05.

## 3. Result

Eighty-four older adults aged 65 to 75 participated in the study, 44 (52.38%) of whom were women and 40 (47.62%) were men. Information on sleep quality, depression level, and nutritional status of older adults is given in Table 1.

**Table 1:** Descriptive data of older adults.

	Older adult N = 84	
	N	%
Sex		
Women	44	52.38
Men	40	47.62
PSQI classification		
Poor sleep quality (PSQI total score ≥ 5)	49	58.33
Good sleep quality (PSQI total score < 5)	35	41.67
GDS-SF classification		
No depressive symptoms (0–4.99 points)	28	33.33
Mild depressive symptoms (5–8.99 points)	22	26.19
Moderate depressive symptoms (9–11.99 points)	14	16.67
Severe depressive symptoms (above 12 points)	20	23.81
MNA-SF classification		
Normally nourished (11–14 points)	20	23.81
At risk (7–10 points)	33	39.29
Malnutrition (<7 points)	31	36.90

% = percentage, GDS-SF = Geriatric Depression Scale-Short Form, MNA-SF = Mini Nutritional Assessment-Short Form, N = total sample size, PSQI = Pittsburgh Sleep Quality Index.

The mean age of the older adults participating in the study was 69.85 ± 2.34 years and the mean BMI was 24.52 ± 2.41 kg/m² (Table 2). The mean NHP, PSQI, GDS-SF, and MNA-SF scores of older adults are given in Table 2.

**Table 2:** Age, BMI, NHP, PSQI, GDS-SF and MNA-SF assessment results of older adults.

	Older adult N = 84
	Mean ± SD
Age (yr)	69.85 ± 2.34
BMI (kg/m²)	24.52 ± 2.41
NHP (point)	92.46 ± 74.84
PSQI (point)	7.07 ± 4.82
GDS-SF (point)	7.07 ± 3.99
MNA-SF (point)	6.48 ± 3.98

BMI = body mass index, GDS-SF = Geriatric Depression Scale-Short Form, kg/m² = kilogram per square meter, MNA-SF = Mini Nutritional Assessment-Short Form, N = total sample size, NHP = Nottingham Health Profile, PSQI = Pittsburgh Sleep Quality Index, SD = standard deviation.

Multiple linear regression analysis results showed that 71.9% (Adjusted *R*² = .719 *P* < .001) of the NHP score variance was explained by PSQI, GDS-SF, and MNA-SF. Additionally, sleep quality (Beta = .440, *t* = 4.861, *P* < .001), depression (Beta = 0.902, *t* = 4.273, *P* < .001), and nutritional status (Beta = -.463, *t* = −2.302, *P* = .024) were found to have significant effects on quality of life (Table 3).

**Table 3:** Multiple linear regression analysis results for NHP.

	Older adults N = 84						
	NHP						
	Unstandardized coefficients		Standardized coefficients	*t*	*P*	Adjusted *R*^2^	*P*
	B	SE	Beta				
Constant	-131.913	50.855		-2.592	.011		
PSQI	6.833	1.406	0.440	4.861	<.001	.719	<.001
GDS-SF	16.904	3.956	0.902	4.273	<.001		
MNA-SF	-8.712	3.784	-0.463	-2.302	.024		

B = unstandardized coefficient, Beta = standardized coefficient, GDS-SF = Geriatric Depression Scale-Short Form, MNA-SF = Mini Nutritional Assessment-Short Form, N = total sample size, NHP = Nottingham Health Profile, PSQI = Pittsburgh Sleep Quality Index, *R*² = coefficient of determination; multiple linear regression analysis, SE = standard error.

*P* = significance level, *P* < .05.

## 4. Discussion

In this study, the quality of life, sleep quality, depression level, and malnutrition status of older adults were assessed using videoconferencing-based evaluations. Prevalence findings showed that 58.3% of the participants had poor sleep quality, 40.5% exhibited moderate to severe depressive symptoms, and 36.9% were at risk of malnutrition. According to the results of multiple linear regression analysis, the model including these 3 predictors explained 71.9% of the total variance in quality of life scores (adjusted *R*² = .719), indicating that sleep quality, depression, and malnutrition have a substantial impact on quality of life. When standardized regression results were examined, depression level emerged as the strongest predictor of lower quality of life (Beta = .902) followed by malnutrition (Beta = –.463) and sleep quality (Beta = .440). The positive beta for sleep quality reflects the inverse scoring structure of the scale, where higher scores indicate worse sleep quality. In contrast, the negative beta for malnutrition indicates that a higher risk of malnutrition is associated with lower quality of life. These findings highlight the multidimensional structure of quality of life in older adults and the important role of both psychological and physiological factors. In particular, the high beta value for depression suggests not only statistical significance but also clinical relevance. Therefore, addressing depressive symptoms should be prioritized in interventions aimed at improving quality of life in this population.

### 4.1. Sleep quality, depression, and nutritional status assessment results

Sleep problems in older adults are closely related to the physiological, psychological, and social effects of aging and are one of the most common health problems in this population.^[28]^ Poor sleep quality affects 40% to 70% of individuals worldwide, and some studies report prevalence rates exceeding 50%, particularly among older adults.^[29]^ In a meta-analysis involving community-dwelling individuals aged 60 years and older, the rate of poor sleep quality measured by the PSQI was reported to be 41%.^[30]^ This prevalence necessitates systematic assessment and regular monitoring of sleep status. In recent years, there has been a marked increase in the use of video conferencing, mobile health applications, and other telehealth-based approaches to support continuous monitoring of health status.^[31]^ In a comprehensive review conducted by Grotto et al, it was reported that mobile health technologies can be used to improve sleep quality in older adults.^[32]^ Similarly, in another study, in a 6-week virtual intervention study called “Sleep Education for Elders Program,” significant improvements in sleep quality indicators were obtained as a result of group sessions conducted via video conferencing under the guidance of a health educator.^[33]^ Furthermore, in a randomized controlled trial conducted with individuals with chronic insomnia, cognitive behavioral therapy delivered via videoconferencing was found to be similarly effective as face-to-face interventions in improving sleep quality.^[34]^ This finding suggests that video-based applications may be not only technically feasible but also a clinically effective intervention tool. In the current study, 56.3% of the older adults assessed via video conferencing had poor sleep quality. This rate is similar to the findings obtained previously with different methods. These results suggest that videoconference-based assessment methods may offer a practical, accessible, and potentially effective alternative for monitoring and assessing sleep quality.

Depression is a multidimensional mental health problem that is common in older adults and negatively affects quality of life due to many factors such as biological changes that occur with aging, chronic diseases, loss of social roles, loneliness, and economic difficulties.^[35]^ Although different results have been reported in the literature regarding the prevalence of depression in older adults, a comprehensive meta-analysis conducted in 2022 reported that the average prevalence of depression in individuals aged 60 years and older was 28.4%.^[36]^ Another meta-analysis of global data reported that 30.2% to 40.4% of older adults had depression, with an average prevalence of 35.2%.^[37]^ In recent years, videoconferencing and other telehealth-based approaches have been increasingly adopted in the management and assessment of depression in older adults.^[38]^ In a randomized controlled trial conducted by Choi et al, problem-solving therapy delivered via videoconferencing to low-income older adults living at home was shown to significantly reduce depressive symptoms.^[39]^ A systematic review and meta-analysis found that telehealth-based psychosocial interventions were effective in alleviating symptoms of depression in older adults.^[40]^ Another study comparing telephone and face-to-face interventions for the assessment of depression reported a high level of consistency in the results of both methods.^[25]^ In a randomized controlled trial conducted with cardiac patients, it was reported that the effects of telerehabilitation on depression, anxiety, and quality of life were similar when compared with traditional face-to-face rehabilitation, and there was no statistically significant difference between the 2 methods.^[41]^ These findings support that depression can be reliably assessed by telehealth methods. In our study, 40.3% of the older adults evaluated by videoconferencing had moderate or higher levels of depressive symptoms. This rate is consistent with the prevalence of depression reported in the existing literature and indicates that depression is still an important public health problem among older adults. Psychosocial assessment and support offered through videoconferencing can provide an inclusive and sustainable alternative, especially for individuals who have difficulty accessing traditional health services. In today’s increasingly digitalized health services, such approaches can be considered as an effective component of holistic intervention models to be developed to monitor and support the mental health of older adults.

Malnutrition is a critical problem affecting health, functional independence, and quality of life in older adults.^[42]^ In the literature, the prevalence of malnutrition in community-dwelling older adults has been reported to be highly variable. In a comprehensive meta-analysis conducted by Salari et al, the global malnutrition rate was reported to be 18.6%; however, the study emphasized that this rate varied between 10% and 40% depending on the country and reached higher levels of 35.7% in Africa.^[43]^ In a study conducted in older adults living in nursing homes in Mexico, the malnutrition rate was reported to be 21.1%.^[44]^ In another study involving urban and rural older adults in Ethiopia, the prevalence ranged between 23% and 46%.^[45]^ Following the identification of this high risk profile in older adults, research on videoconferencing and telehealth-assisted nutrition interventions has begun to proliferate. In recent years, video conferencing and telehealth methods have been increasingly used in this field as well.^[46]^ In a pilot study conducted by Gomes et al, individual nutrition counseling was provided to food insecure older adults through a 12-week home-based e-health application, which resulted in significant improvements in energy intake, physical function, and quality of life.^[47]^ A systematic review and meta-analysis by Trivedi et al showed that telehealth-based nutrition interventions had significant ameliorative effects on LDL cholesterol, sodium intake, and systolic blood pressure in older adults. In addition, these interventions were reported to be highly feasible and acceptable by individuals.^[48]^ Furthermore, in a randomized controlled trial conducted with individuals with type 2 diabetes, telenutrition education delivered via video conferencing was shown to be similarly effective to face-to-face interventions in terms of glycemic control.^[49]^ In this study, malnutrition was detected in 36.9% of older adults assessed by videoconferencing. This rate is in line with previously reported data and shows that nutritional deficiencies are still prevalent in the older adults. Especially in groups with limited access to health systems, remote monitoring of nutritional status can offer a significant advantage in terms of both early risk detection and timely intervention. Accordingly, video-based assessment and counseling systems should be considered not only as a technological solution but also as a functional tool that can balance the unequal distribution of health services.

### 4.2. The effect of sleep quality, depression, and nutritional status on quality of life

Quality of life in older adults is a multidimensional concept shaped by the interaction of physical, mental and social health components. In this context, sleep quality, depression level, and nutritional status are among the main factors determining quality of life. In the literature, there are many studies addressing the relationship between these 3 variables and quality of life separately. In regression analyses on sleep quality, it was reported that the effect of sleep efficiency and negative beliefs about sleep on quality of life was 24%.^[50]^ In a study conducted with older adults, it was determined that poor sleep quality increased the likelihood of being in the lowest quartile of quality of life by approximately 2 times.^[51]^ In a longitudinal analysis conducted in Portugal and based on SHARE data, it was revealed that 42.1% of the variance in quality of life that changes with age could be explained and the highest contribution to this variance belonged to the level of depression.^[52]^ Similarly, Zhou et al’s structural equation model findings indicate that depression has a strong and significant direct effect on quality of life.^[53]^ Nutritional status is also an important determinant of quality of life. A meta-analysis concluded that nutrition has a greater impact on quality of life than exercise and demographic variables.^[54]^ In another study, nutritional status was found to be a very important determinant of quality of life.^[55]^ The findings of this study show that depression level, nutritional status and sleep quality together explain 71.9% of the variance in quality of life according to multiple linear regression analysis. When the effect sizes were analyzed, it was observed that the strongest determinant of QoL was depression level, followed by nutritional status and sleep quality, respectively. The fact that depression has the highest effect size clearly reveals the central role of psychological state on the quality of life of older adults. Although all 3 factors were found to be significant contributors, the prominence of depression underscores the need to prioritize mental health in clinical practice. Nutrition and sleep should also be addressed as essential and complementary domains in holistic strategies aimed at enhancing quality of life. In conclusion, supporting mental health, improving nutritional status, and increasing sleep quality should be addressed with a holistic approach in health practices aimed at improving the quality of life of older adults.

This study has some limitations. The research was conducted with a one-group design and it was not possible to compare the findings obtained with the video conferencing method with other assessment methods due to the absence of a control group. In addition, short forms were preferred as assessment tools. This choice was influenced by the need to minimize the attention span and assessment time of older adults. However, short forms may limit the detailed analysis of some sub-dimensions. In addition, the fact that the sample consisted of individuals living in only 1 city center limits the generalizability of the results to different socio-cultural and regional groups. In future studies, the preference for designs in which different methods are compared and more detailed analyses can be conducted will strengthen the scope and interpretability of the findings.

## 5. Conclusion

This study investigated the key determinants of quality of life among older adults using a videoconference-based assessment approach. The findings revealed that 58.3% of participants had poor sleep quality, 40.5% exhibited moderate to high levels of depressive symptoms, and 36.9% were found to be malnourished. According to the multiple linear regression analysis, sleep quality, depression level, and nutritional status together explained 71.9% of the variance in quality of life, with depression level identified as the most significant predictor. These results indicate that depression, sleep quality, and nutritional status form not only individually important but also collectively powerful and interrelated determinants of quality of life in older adults. Therefore, interventions aiming to improve quality of life should be designed with a multidimensional approach that simultaneously addresses mental well-being, nutritional adequacy, and sleep regulation. Moreover, in line with the principles of community-based rehabilitation, enhancing access to digital health services, improving digital literacy, and implementing public awareness campaigns may facilitate early detection of health risks and support healthy aging. These strategies are consistent with the goals of the World Health Organization’s Decade of Healthy Ageing (2021–2030).

## Acknowledgments

The authors thank all the older adults who participated in the study.

## Author contributions

**Conceptualization:** Özgün Elmas, Mustafa Cemali, Özge Cemali.

**Data curation:** Özgün Elmas, Mustafa Cemali, Özge Cemali.

**Formal analysis:** Mustafa Cemali, Özge Cemali.

**Funding acquisition:** Özgün Elmas, Mustafa Cemali, Özge Cemali.

**Investigation:** Özgün Elmas, Mustafa Cemali, Özge Cemali.

**Methodology:** Özgün Elmas, Mustafa Cemali, Özge Cemali.

**Project administration:** Özgün Elmas, Mustafa Cemali.

**Resources:** Özgün Elmas, Mustafa Cemali.

**Software:** Özgün Elmas, Mustafa Cemali, Özge Cemali.

**Supervision:** Özgün Elmas, Özge Cemali.

**Validation:** Özgün Elmas, Mustafa Cemali, Özge Cemali.

**Visualization:** Özgün Elmas, Özge Cemali.

**Writing – original draft:** Özgün Elmas, Mustafa Cemali, Özge Cemali.

**Writing – review & editing:** Özgün Elmas, Mustafa Cemali, Özge Cemali.
